# Assembled pH-Responsive Gastric Drug Delivery Systems Based on 3D-Printed Shells

**DOI:** 10.3390/pharmaceutics16060717

**Published:** 2024-05-27

**Authors:** Haoye Bei, Pingping Zhao, Lian Shen, Qingliang Yang, Yan Yang

**Affiliations:** College of Pharmaceutical Science, Zhejiang University of Technology, Hangzhou 310014, China; 2112107049@zjut.edu.cn (H.B.); zhaopingping69@163.com (P.Z.); shenlian2021@zjut.edu.cn (L.S.); qyang@zjut.edu.cn (Q.Y.)

**Keywords:** gastric drug delivery systems, assembled modular units, 3D printing, pH response, self-regulation

## Abstract

Gastric acid secretion is closely associated with the development and treatment of chronic gastritis, gastric ulcers, and reflux esophagitis. However, gastric acid secretion is affected by complex physiological and pathological factors, and real-time detection and control are complicated and expensive. A gastric delivery system for antacids and therapeutics in response to low pH in the stomach holds promise for smart and personalized treatment of stomach diseases. In this study, pH-responsive modular units were used to assemble various modular devices for self-regulation of pH and drug delivery to the stomach. The modular unit with a release window of 50 mm^2^ could respond to pH and self-regulate within 10 min, which is related to its downward floatation and internal gas production. The assembled devices could stably float downward in the medium and detach sequentially at specific times. The assembled devices loaded with antacids exhibited smart pH self-regulation under complex physiological and pathological conditions. In addition, the assembled devices loaded with antacids and acid suppressors could multi-pulse or prolong drug release after rapid neutralization of gastric acid. Compared with traditional coating technology, 3D printing can print the shell layer by layer, flexibly adjust the internal and external structure and composition, and assemble it into a multi-level drug release system. Compared with traditional coating, 3D-printed shells have the advantage of the flexible adjustment of internal and external structure and composition, and are easy to assemble into a complex drug delivery system. This provides a universal and flexible strategy for the personalized treatment of diseases with abnormal gastric acid secretion, especially for delivering acid-unstable drugs.

## 1. Introduction

Gastric pH is related to gastric acid secretion and is affected by both physiological and pathological factors. The basal acid output, which estimates resting secretion, is about 10 mmol H^+^ per hour in men and 5 mmol H^+^ per hour in women. The acid output may vary from one hour to the next in an individual, and the median fasting pH is 1.5 with a normal range of 0.3–2.9 [1]. Increased acid secretion might cause gastroesophageal reflux disease, while decreased acid secretion is related to chronic atrophic gastritis, dyspepsia, and infections. During the treatment of stomach diseases, commonly used clinical methods for detecting gastric acid levels, such as nasogastric intubation [2] or Heidelberg capsule [3], are complicated and expensive to perform. Generally, antacids are administered in combination with acid inhibitors and gastric mucosal protectants to neutralize excess stomach acid, either at the onset of symptoms (heartburn) or before (after meals or before sleep). Drug delivery systems (DDSs), such as gastric floating [4] and gastric adhesion [5], are widely used to prolong efficacy, including inhibiting acid secretion and repairing the gastric mucosa. However, these treatment strategies cannot respond quickly to low pH in the stomach or reduce overtreatment under stomach-neutral conditions. 

DDSs with pH-responsiveness provide on-demand drug delivery in complex and variable acidic environments. For example, in situ hydrogels with gastric acid pH responsiveness for the sustained release of baicalin have been produced to alleviate oxidative stress damage in gastric ulcer disease [6]. This in situ hydrogel system has promising applications in the sustained oral release of acid-unstable drugs [7]. Another pH-responsive hydrogel bead was synthesized based on sodium alginate and carboxymethyl chitosan, and was promoted by hydrogen bonds [8]. The swelling ratio of the beads and their protein-release profiles were pH-dependent, which could prevent premature protein release into the gastric environment. An injectable pH-responsive self-healing adhesive hydrogel based on acryloyl-6-aminocaproic acid (AA) and AA-g-N-hydroxy succinimide was developed [9]. The hydrogels exhibited suitable gelation time, autonomous and efficient self-healing capacity, hemostatic properties, and good biocompatibility. However, multiple pH-responsive DDSs that can be used in complex and changeable gastric acid environments have been less widely reported. 

Recently, stimulus-responsive DDSs, particularly those with multiple responses to pH changes in the microenvironment, have been developed to achieve multiple smart responses [10]. A glucose-responsive insulin delivery system was developed using a pH-sensitive peptide hydrogel loaded with glucose oxidase, catalase, and insulin as the carrier [11]. Glucose is converted to gluconic acid by glucose oxidase, and the resulting low pH causes the disassembled hydrogel to release insulin. A pH-triggered, size-transformable, self-assembling chimeric peptide nanofiber was developed to treat drug-resistant bacteria [12]. The chimeric peptide was transformed into nanoparticles in the acidic biofilm (pH 5.0) and penetrated the bacterial biofilms to kill the bacteria. These stimuli-responsive DDSs based on the microenvironment (i.e., pH, temperature, and enzymes) have implications for the smart delivery of drugs in the stomach.

Eudragit^®^ EPO (EPO) is dissolved in the acidic solution (pH < 5) because its basic site contains tertiary amine groups, which are ionized in acids. Therefore, EPO is commonly used as a coating material in oral formulations for taste-masking or gastric immediate release [13]. Besides this, EPO could be used to prepare nanoparticles and microcapsules to improve the solubility of insoluble drugs in the stomach. For example, valsartan-loaded EPO solid dispersion microparticles were prepared using a spray-drying technique [14]. The drug released from solid dispersion in 15 min at pH 1.2 was almost 100%, while the drug released from free powder at pH 1.2 was less than 1%. Besides this, spray-drying encapsulated iron microcapsules were prepared for food fortification [15]. More than 90% iron was released within 30 min under stomach conditions (pH = 1) and less than 15% iron was released in 2 h under ambient conditions (pH = 7).

3D printing can easily regulate the geometry [16] and internal structure [17] of preparations, thus customizing drug release behavior. For example, various types of drug-polyvinyl alcohol (PVA)/PVA and drug-PVA/polylactic acid (PLA) composite tablets have been 3D-printed [18]. Drug release profiles can be tailored by changing the surface area of the exposed drug component and the thickness of the PVA filler. However, the lack of suitable pharmaceutical polymers for printing filaments limits the promotion of fused deposition modeling (FDM) in personalized medicine [19]. Therefore, loading active ingredients into FDM-printed modular devices is a clinically feasible way to customize the drug release behavior (i.e., immediate, pulsed, and sustained). For example, immediate- and extended-release liquid capsule shells were FDM-printed using Eudragit^®^ EPO (EPO) and RL to load a drug solution or suspension [20]. Colon-specific tablet shells were FDM-printed using Eudragit^®^ FS100 and PLA to fill the drug-loaded hydrogels [21]. Tablet-shaped devices were filled with riboflavin tablets to produce floating gastric systems [22]. Devices with various net- and air-filled chambers were FDM-printed to regulate drug release and floating behavior. Similarly, capsule-shaped floating devices were FDM-printed to control the release and gastric retention of domperidone tablets [23]. Increasing the cap thickness of the devices could increase the total floating time, whereas decreasing the hole size on the devices could enable the sustained release of drugs. In addition, unique suppository shells were FDM-printed to load progesterone into a tailed vaginal suppository [24]. The drug release profiles were related to the position, number, and diameter of the holes in the suppository shells. 

3D printing offers an easy method to produce personalized medicines with multiple units for combination therapy and programmed drug release. For example, a polypill with immediate- and sustained-release compartments was extrusion-printed to independently deliver five cardiovascular drugs with defined release profiles [25]. A dual-compartmental dosage unit was FDM-printed to physically isolate and modulate the release profile of antituberculosis drugs, such as rifampicin and isoniazid [26]. Considering the efficiency and flexibility of personalized medicine, FDM-printed universal modular units can be assembled for on-demand drug delivery. For example, capsules and disc-shaped tablets were fabricated by assembling a surface-eroding drug-loaded matrix and a blank matrix into an impermeable coating [27]. The tablets were customized on demand using 3D-printed molds with different shapes and sizes because the release profile was highly related to the shape. Similarly, using FDM-printed capsule shells as modular units, various multi-compartment devices can be assembled for drug delivery. Can-capsules and modular Super-H capsules with concentric compartments [28] can deliver drugs to the small intestine or deliver two different drugs. Capsular devices with two compartments [29] could deliver various active ingredients in a tunable dual-pulse release pattern. Capsular devices with three compartments [30] were assembled, and the performance of each compartment (immediate, delayed, or pulsatile release) depended only on the composition and thickness of the wall. In addition, modular devices assembled for gastric retention have been developed for human immunodeficiency virus antiretroviral therapy [31] and malaria elimination [32]. The star-shaped dosage, consisting of six drug-loaded arms, prevented passage through the pylorus and enabled ultra-long gastric residence. Another assembled modular device for implants was developed to realize zero-order pulsatile release, dose-dense therapy, and combination therapy by programming the inner-layer structural design on the microscale [33].

In this study, using EPO as the pH-responsive polymer and sodium bicarbonate (NaHCO_3_) as the antacid drug, modular units were prepared based on the FDM-printed insoluble shells. The effects of the release window area and core composition on the pH response speed and mechanism were investigated. The PVA connector and floating chamber were designed to ensure that the modular units remained floating with the release window downward and detached in sequence on demand. Using acid suppressor famotidine (FAM) as another drug combination, modular units were assembled into devices with multiple pH responses (automatic acid neutralization and drug delivery three times). By simulating various gastric acid secretion conditions in vitro, the response performance and personalized therapeutic prospects in complex and varied gastric environments were evaluated.

## 2. Materials and Methods

### 2.1. Materials

EPO (No. G171231674) was purchased from Evonik Rohm Co., Ltd. (Darmstadt, Germany). Triethyl citrate (No. 181126) was obtained from Fengyuan Pharmaceutical Co., Ltd. (Hefei, China). Tween 80 was purchased from Sinopharm Group Chemical Reagent Co., Ltd. (Shanghai, China). NaHCO_3_ (No. 20230524) was purchased from Kangqiong Biomedical Technology Co., Ltd. (Wuhan, China). Soluble starch (No. 221204) was purchased from Anhui Sunhere Pharmaceutical Excipients Co., Ltd. (Huainan, China). Low-substituted hydroxypropyl cellulose (L-HPC, No. 5111432) was purchased from Shin-Estu Chemical Co., Ltd. (Tokyo, Japan). FAM (No. 20220309) was purchased from Hubei Ricentik Biotechnology Co., Ltd. (Xiangyang, China). Hydroxypropyl methylcellulose (HPMC, K100LV, No. SH234195) was purchased from Shanghai Colorcon Coating Technology Co., Ltd. (Shanghai, China). PVA and PLA filaments were purchased from Polymaker Co., Ltd. (Shanghai, China).

### 2.2. Preparation of Modular Units

**Figure 1:**
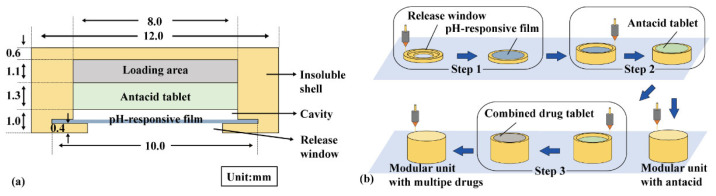
Modular unit: (**a**) components and (**b**) preparation process.

A modular unit (Figure 1a) consists of an insoluble shell (yellow) with a release window, a pH-responsive film (blue), and an antacid tablet (green). For multiple-drug delivery in combination therapy, a combined drug tablet (FAM immediate- or sustained-release tablet) was added in the loading area (gray). The insoluble shell was sliced using Cura 5.2.1 software with a layer height of 0.1 mm, and then printed using a PLA filament in an FDM printer (TL-D3 Pro, Tenglong, China) at 210 °C and 50 mm/s. As shown in Figure 1b, the insoluble shell was printed from bottom to top, and the pH-responsive film was manually added above the release window after printing the fourth layer and sealed using adhesive (Step 1). The antacid tablets and combined drug tablets were manually added during printing (Steps 2–3). 

EPO, triethyl citrate, and tween 80 were mixed at a ratio of 60:5:2, and methylene blue was added to observe film integrity. A polymer solution (33.5% *w*/*v*) was prepared using 95% ethanol as the solvent and then spread on a Teflon plate with a thickness of 1.0 mm. After drying at 25 °C for 8 h, the pH-responsive film was removed and cut into a circle with a diameter (*Φ*) of 10.0 mm and a thickness (*H*) of approximately 0.15 mm. 

NaHCO_3_, soluble starch, and L-HPC were mixed in a ratio of 5:4:1 and then pressed into 100 mg antacid tablets (*Φ* = 8.0 mm, *H* approx. 1.3 mm, hardness approx. 40 N). FAM, soluble starch, and L-HPC were mixed in a ratio of 5:13:2 and then pressed into an 80 mg immediate-release tablet (*Φ* = 8.0 mm, *H* approx. 1.2 mm, hardness approx. 40 N). FAM, soluble starch, and HPMC were mixed in a ratio of 5:11:4 and then pressed into an 80 mg sustained-release tablet (*Φ* = 8.0 mm, *H* approx. 1.2 mm, hardness approx. 40 N).

### 2.3. The pH Response and Self-Regulation of Modular Units

Modular units were placed in 50 mL of simulated gastric fluid (HCl solution, pH = 2.00, 37 °C), and the pH value was recorded using a pH meter. A sample of 1.0 mL was taken at a specific time, diluted 100 times using 0.01 N HCl, and alkalized with 0.25 mL of ammonium hydroxide (28%). The Na^+^ concentration was measured using a Na^+^ concentration meter, and the cumulative NaHCO_3_ release (*Q_NaHCO3_*) was calculated. The release rate of NaHCO_3_ (*V_NaHCO3_*) was calculated based on the increase in *Q_NaHCO3_* at adjacent time points, and the maximum release rate of NaHCO_3_ (*V_m-NaHCO3_*) was recorded. The time required for the modular units immersed in the medium for the antacid tablets to disintegrate is defined as the pH-responsive lag time (*T_rl_*). The time taken for the pH to rise from 2.00 to 5.50 is defined as the pH self-regulation duration (*T_sr_*).

### 2.4. Preparation of Assembled Devices

**Figure 2:**
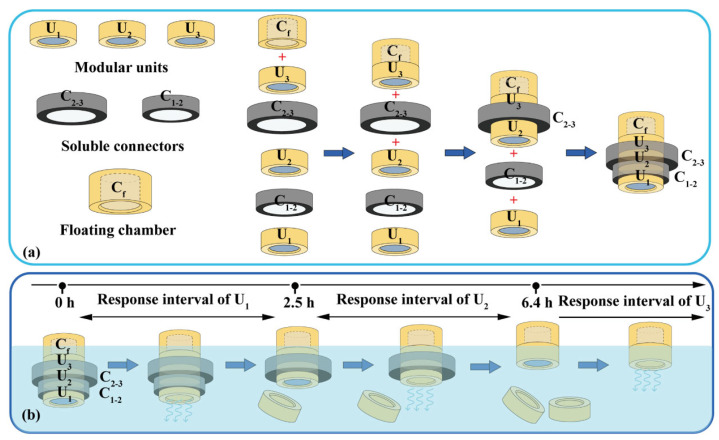
Typical assembled devices with three units: (**a**) assembly process and (**b**) dissolution process. U_1_, U_2_, U_3_: the 1st, 2nd and 3th modular units. C_1–2_: soluble connector connected the 1st and 2nd units. C_2–3_: soluble connector connected the 2nd and 3th units. C_f_: floating chamber.

Assembled devices consist of modular units, soluble connectors, and floating chambers (Figure 2a). A typical soluble connector is a ring with an inner diameter of 12.0 mm, a height of 4.0 mm, and a wall thickness of 0.8–2.4 mm. The soluble connector was FDM-printed using a PVA filament at 200 °C and 40 mm/s. A typical floating chamber is a cylinder with an internal cavity and variable volume for density adjustment. The floating chamber was FDM-printed using a PLA filament at 210 °C and 50 mm/s. 

A typical device with three units was assembled as illustrated in Figure 2a. The floating chamber (C_f_) was attached to the unit (U_3_) via 3D printing or using adhesive. Units U_2_ and U_3_ were connected using connectors C_2–3_; units U_1_ and U_2_ were connected using connectors C_1–2_, and then sealed using adhesive. The volume of the floating chambers was designed to adjust the density (≈0.95 g/cm^3^) to ensure stable floating. 

### 2.5. The pH Response and Drug Delivery of Assembled Devices

The assembled devices were placed in 50 mL of distilled water, and the pH value was recorded. To simulate rapid acid secretion, an appropriate amount of 0.1 N HCl solution was rapidly added to reduce the pH from neutral to 2.00. Then, 50 μL of 0.1 N HCl solution was added to simulate slow acid secretion at 0.5 h intervals. The typical dissolution process is illustrated in Figure 2b, where the modular units showed pH-responses and drug releases, and detached sequentially. The sample (1.0 mL) was collected at a specific time and assayed at 266 nm using a UV-vis spectrophotometer (759CRT, Juchuang, China). Cumulative FAM release (*Q_FAM_*) was calculated using a standard curve (*A* = 0.0299*C* − 0.0305, *R* = 0.9998), where *A* is the absorption and *C* is the FAM concentration. *Q_NaHCO3_* and *V_NaHCO3_* were determined as described in Section 2.3.

## 3. Results and Discussion

### 3.1. pH Response and Self-Regulation of Modular Units

**Figure 3:**
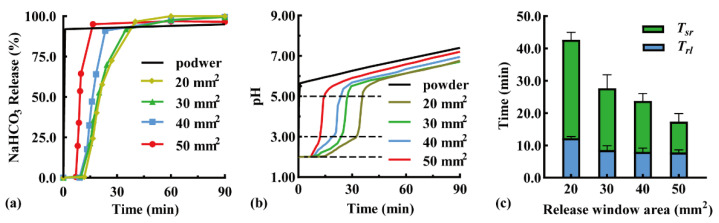
Effect of release window area of modular units on (**a**) typical NaHCO_3_ release curves, (**b**) typical pH value curves, (**c**) pH-responsive lag time (*T_rl_*), and pH self-regulation duration (*T_sr_*) (n = 3).

The modular unit was immersed downward in the simulated gastric fluid, and the effect of the release window area on pH-responsive drug release behavior was investigated. Typical NaHCO_3_ release curves are shown in Figure 3a because the sampling time varied with pH response. When the release window increased from 20 to 50 mm^2^, the *T_rl_* was shortened from 11.8 to 7.0 min, while the complete release time was shortened from 40.2 min (*Q_NaHCO3_* = 96.7%) to 16.5 min (*Q_NaHCO3_* = 95.1%). A larger release window promoted contact between the pH-responsive film and acidic medium, thus accelerating film dissolution and NaHCO_3_ release. In addition, the particles that disintegrated from the antacid tablets were more easily discharged through the expanded release window. A similar result has been reported in the literature [34], in which the amount of drug released in 90 min increased from >60% to >80% with a larger release window diameter from 0.1 to 0.3 mm. 

An antacid tablet loaded with 50 mg NaHCO_3_ was filled in the modular unit. The effect of the release window area on the pH self-regulation ability was investigated (Figure 3b). Within a low pH range of 2.00–3.00, the pH self-regulation increased from 0.047 to 0.18 pH units/min with an increased window area from 20 to 50 mm^2^, because NaHCO_3_ was released faster through a larger release window. Within a medium pH range of 3.00–5.50, a small amount of NaHCO_3_ being released rapidly increased the pH value; as a result, the release window area had little effect. Within a high pH range of 5.50–7.50, pH increased slowly, which was not related to the NaHCO_3_ release. This was demonstrated by the addition of 50 mg of NaHCO_3_ powder to the simulated gastric fluid as a control (black line). After the NaHCO_3_ powder was completely dissolved, the pH of the solution further increased from 5.50 to 7.50 within 90 min. Similarly, the pH of a 1 N NaHCO_3_ solution increased from 5.5 to 8.3 after 10 h [35], which may be a consequence of the spontaneous loss of CO_2_ from the solution [36].

The effects of the release window area on the pH response and self-regulation are compared in Figure 3c. With an increase in the release window area from 20 to 50 mm^2^, the *T_rl_* reduced from 12.2 ± 0.5 to 7.8 ± 0.8 min, while the *T_sr_* reduced from 30.5 ± 2.4 to 9.0 ± 2.6 min, suggesting a negative correlation relationship. Therefore, fast pH response and self-regulation can be achieved by maximizing the release window area of the unit. 

**Figure 4:**
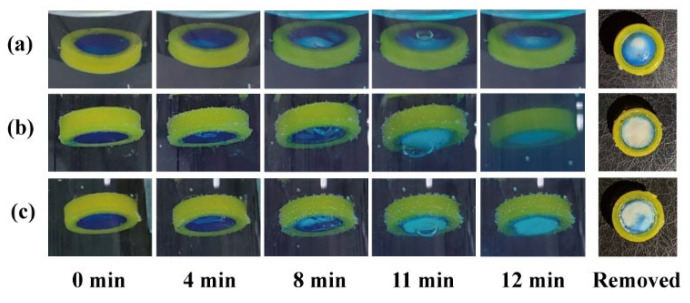
The pH response process and remaining film of modular units: (**a**) containing an antacid tablet immersed upward and (**b**) downward; (**c**) containing a starch tablet immersed downward.

The effects of the release window orientation on the pH response process were compared using a release window area of 50 mm^2^. When the modular unit was immersed upwards in the simulated gastric fluid (Figure 4a), the pH-responsive film slowly expanded and deformed within 8 min. The film remained intact (with a small bubble inside) at 11 min and ruptured at 12 min. The unit was quickly removed after 12 min, and the remaining pH-responsive film (blue) accounted for 4/5 of the window area. The results demonstrate that the sealing was stable in the medium. Pharmaceutical adhesives, such as cellulose acetate acetone solution [37], should be further investigated for clinical safety. 

When the response unit was immersed downward in the medium (Figure 4b), the pH-responsive film expanded and deformed more significantly within 8 min. The film remained intact (with a large bubble inside) at 11 min, and ruptured at 12 min. A small pH-responsive film remained on the quick-removal unit at 12 min. Small bubbles floating upward struggle to discharge through the downward release window; therefore, they merge into large bubbles, accelerate film rupture, and promote medium inflow and tablet disintegration. Instead of an antacid tablet (50% NaHCO_3_), a starch tablet (0% NaHCO_3_) was filled into the modular unit to investigate whether the gas production of NaHCO_3_ could promote film rupture. As shown in Figure 4c, the modular units containing 0% NaHCO_3_ could also generate bubbles, which might come from the air contained in the core and shell. Compared with the results in Figure 4b, the bubble at 11 min in Figure 4c was smaller, while the remaining film area at 12 min was larger (approx. 1/3). This suggests that both gas production and the downward release window contributed to the rapid pH response of the modular units. 

### 3.2. Connection Pattern and Floating Behavior of the Devices with Two Units

**Figure 5:**
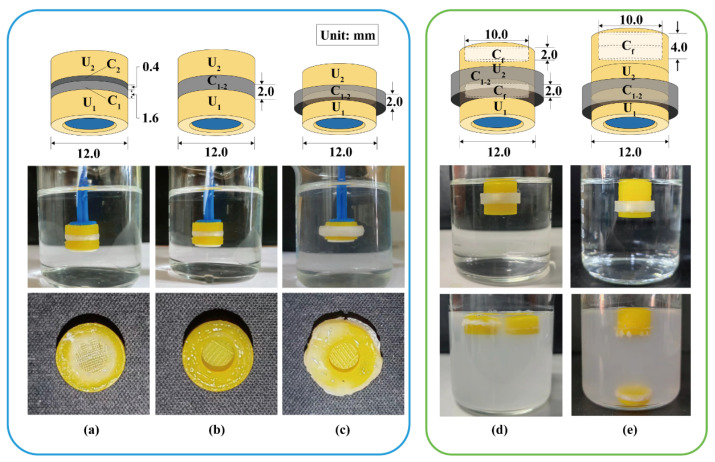
Detachment behavior of assembled units: (**a**) a double-layer cylinder vertically connected, (**b**) a ring vertically connected, and (**c**) a ring laterally connected. The floating behavior of assembled units with a floating chamber at the top of (**d**) each unit or (**e**) assembled units. U_1_, U_2_: the 1st and 2nd modular units. C_1_, C_2_: soluble connector with a fill density of 10% and 100%. C_1–2_: soluble connector connected to the 1st and 2nd units. C_f_: floating chamber.

Two modular units were assembled using different soluble connectors to investigate the detachment behavior in simulated gastric fluid. As shown in Figure 5a, a double-layer cylinder was used to connect the two units. The loose layer C_1_ (*H* = 1.6 mm) with a fill density of 10% adhered to the U_1_ such that U_1_ could completely detach from the device after 2.5 h. The dense layer C_2_ (*H* = 0.4 mm) with a fill density of 100% adhered to the U_2_ to ensure the U_2_ remained “silent” in the simulated gastric fluid until the U_1_ detached. Although C_2_ could effectively isolate the pH-responsive film from the acidic medium, the dense layer dissolved slowly, and the remaining PVA was blocked in the release window, reducing the response speed. As shown in Figure 5b, a ring (*H* = 2.0 mm) with inner and outer diameters of 8.0 and 12.0 mm was used to connect the two units vertically. U_1_ completely detached after 3.0 h and no polymer residue remained in the release window of U_2_. As shown in Figure 5c, a ring (*H* = 2.0 mm) with inner and outer diameters of 12.0 and 16.0 mm was used to connect the two units laterally. U_1_ completely detached after 5.0 h and no polymer residue remained in the release window of U_2_. To maintain the release window area of U_2_ and minimize the height of the assembled device, the connection pattern in Figure 5c is preferred.

A ring (*H* = 4.0 mm) with inner and outer diameters of 12.0 and 14.4 mm was used as the soluble connector to laterally connect two units. As shown in Figure 5d, two flotation chambers with a small internal cavity (*H* = 2.0 mm) were added to the top of units U_1_ and U_2_. The assembled units floated stably in the medium, whereas U_1_ detached after 2.6 h and remained afloat. As shown in Figure 5e, a flotation chamber with a large internal cavity (*H* = 4.0 mm) was added to the top of the unit U_2_. The gravity center was lower than the buoyancy center and located on the buoyancy action line (central axis). Therefore, the assembled units could stably float downward in the medium, whereas U_1_ detached after 2.5 h but sank to the bottom. The design in Figure 5e is more conducive to the sequential detachment, sinking, and discharge of each unit from the device. Similarly, 3D-printed tablets containing a top air chamber (density < 0.9 g/cm^3^) could float downward in the medium for a long time [38]. A special PVA structure can be added to promote the disintegration and discharge of the flotation chamber [39]. Besides this, adding the cavity structure into U_2_ could eliminate the floating chamber from the assembled device.

**Figure 6:**
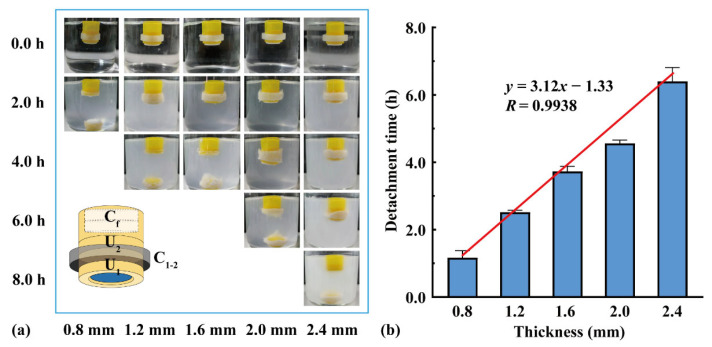
The effect of connector thickness on the assembled units: (**a**) floating process and (**b**) detachment time (n = 3).

Two units were assembled using connectors of different thicknesses, and a flotation chamber (*H* = 4.0 mm) was added to the top. As shown in Figure 6, U_1_ detached from the assembled units after the connector dissolved, and U_2_ continued floating downward. With an increase in the connector thickness from 0.8 to 2.4 mm, the detachment time of U_1_ increased from 1.2 to 6.4 h. The detachment time was positively correlated with the connector thickness (*y* = 3.12*x* − 1.33, *R* = 0.9938), indicating that the detachment time could be adjusted by changing the connector thickness. 

### 3.3. The pH Response and Drug Delivery of the Devices with Three Units

Based on the results in Figure 6b, an assembled device with three units could prepared using soluble connectors with different thicknesses (Figure 2a) to obtain adjustable triple pH response and drug release (Figure 2b). For example, to neutralize excessive acid secretion after meals (i.e., 8:00, 13:00, and 18:00), a triple-unit device can be assembled using C_12_ and C_23_ with thicknesses of 1.2 mm and 2.4 mm, respectively. The detachment times of U_1_ and U_2_ were 2.5 and 6.4 h; therefore, the pH response intervals of U_1_–U_3_ were 0–2.5 h, 2.5–6.4 h, and >6.4 h, respectively. That is, as long as excess gastric acid is secreted within the response time interval, the device can respond and neutralize. The possible time combinations of three times of excessive gastric acid secretion include 0.0–4.0–8.0 h, 0.5–5.0–10.5 h, and 1.0–6.0–9.0 h, which are consistent with diet and medication habits.

**Figure 7:**
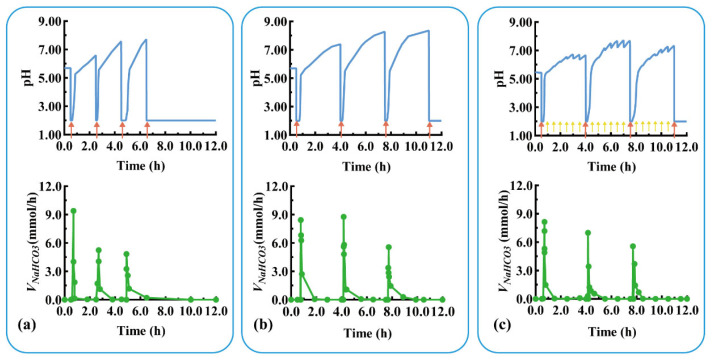
The pH response and NaHCO_3_ release of a typical triple-unit system under different conditions: (**a**) rapid acid secretion at 2 h intervals, (**b**) rapid acid secretion at 3.5 h intervals, and (**c**) rapid acid secretion at 3.5 h intervals combined with slow acid secretion at 0.5 h intervals. Red arrow: rapid acid secretion. Yellow arrow: slow acid secretion. *V_NaHCO3_*: release rate of NaHCO_3_.

Devices with three units were assembled using C_1–2_ and C_2–3_ with thicknesses of 0.8 and 2.0 mm, respectively. The pH response and self-regulation ability of the device were evaluated in a simulated pathological state (mimicking the pH changes of duodenal ulcer) with rapid acid secretion at a short interval of 2 h. As shown in Figure 7a, U_1_ quickly responded to the rapid acid secretion (labeled with a red arrow) at 0.5 h (*T_rl_* = 8.0 min, *T_sr_* = 7.0 min), and the pulsatile released NaHCO_3_ (*V_m-NaHCO3_* = 9.6 mmol/h), regulating the pH from 2.00 back to neutral. After the detachment of U_1_ at 1.0 h, U_2_ was exposed to a neutral medium and ready for the next pH response. For the second acid secretion at 2.5 h, U_2’s_ pH quickly responded (*T_rl_* = 5.5 min, *T_sr_* = 10.0 min) and the pulsatile released NaHCO_3_ (*V_m-NaHCO3_* = 5.4 mmol/h). U_2_ detached after 4.0 h, exposing U_3_ to a neutral medium. Similarly, U_3_ quickly responded to the third acid secretion at 4.5 h (*T_rl_* = 20.0 min, *V_m-NaHCO3_* = 4.8 mmol/h) and self-regulated (*T_sr_* = 10.0 min). For the fourth acid secretion at 6.5 h, no more modular units were available to neutralize the acid, so the pH level remained low.

Devices with three units were assembled using C_1–2_ and C_2–3_ with thicknesses of 1.2 and 2.4 mm, respectively. The pH response and self-regulation ability of the device were evaluated in the physiological state of rapid acid secretion after meals at long intervals of 3.5 h. As shown in Figure 7b, U_1_ quickly responded to the rapid acid secretion (labeled as a red arrow) at 0.5 h (*T_rl_* = 15.0 min, *T_sr_* = 6.7 min), and the pulsatile released NaHCO_3_ (*V_m-NaHCO3_* = 8.4 mmol/h) to neutralize the acid in the medium. After the detachment of U_1_ at 2.5 h, U_2_ was exposed to the medium and prepared for the next pH response. For the second acid secretion at 4.0 h, the U_2_’s pH quickly responded (*T_rl_* = 8.5 min, *T_sr_* = 14.9 min), and the pulsatile released NaHCO_3_ (*V_m-NaHCO3_* = 9.0 mmol/h). U_2_ detached after 6.0 h, exposing U_3_ to the neutral medium. Similarly, the U_3_ quickly responded to the third acid secretion at 7.5 h (*T_rl_* = 10.0 min, *V_m-NaHCO3_* = 5.4 mmol/h) and self-regulated (*T_sr_* = 13.0 min). The assembled units responded to postprandial acid secretion and neutralized excess gastric acid, thereby providing a smart device for treating gastric mucosal damage.

The above three-unit device was further evaluated under a more complex physiological state of rapid acid secretion at 3.5 h intervals combined with slow acid secretion at 0.5 h intervals. As shown in Figure 7c, the device responded sequentially to the three rapid acid secretions (labeled as red arrow) at 0.5, 4.0, and 7.5 h within 10 min, and the pulsatile unit released the antacid to neutralize the medium (pH > 5.5). The device did not respond to the slow acid secretion at 0.5 h intervals (labeled as a yellow arrow); however, the pH value fluctuated around 7.00 due to the buffering effect of NaHCO_3_ released in the medium (discussed in Section 3.1). This indicates that the device is unable to respond in the case of slow gastric acid secretion, preventing the over-regulation of gastric acid. Modular units with different response intervals can be customized to respond to and neutralize a variety of excessive acid secretions. Given the various irregular acid secretions caused by physiological and pathological conditions, personalized modular units can be designed and assembled for combination treatments with multiple drugs.

**Figure 8:**
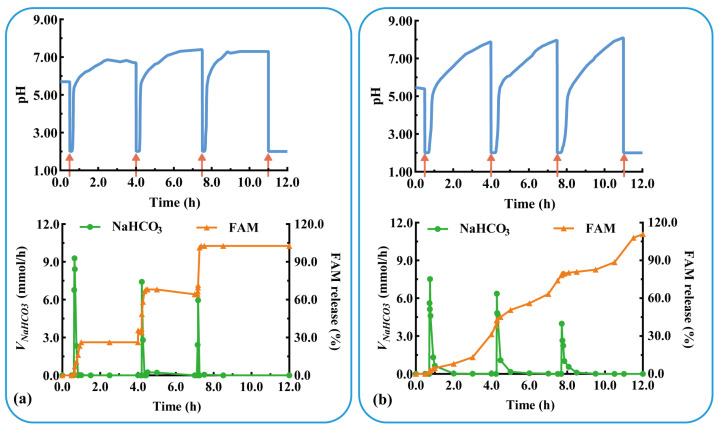
The pH-responsive and multiple drug delivery of a typical triple-unit system containing: antiacid tablets and (**a**) FAM immediate-release tablets or (**b**) FAM sustained-release tablets. *V_NaHCO3_*: release rate of NaHCO_3_. FAM: famotidine.

The acid suppressor FAM was added to the modular unit containing the antacid NaHCO_3_ to evaluate the effect of multidrug delivery in combination therapy. Three units containing FAM immediate-release tablets were assembled and evaluated under physiological conditions of postprandial acid secretion (rapid acid secretion at 3.5 h intervals). As shown in Figure 8a, the assembled units responded rapidly to rapid acid secretion (labeled as a red arrow) at 0.5, 4.0, and 7.5 h (*T_rl_* = 7.0, 9.0, and 6.0 min). NaHCO_3_ was released in a pulsatile manner (*V_m-NaHCO3_* = 7.0, 9.0, and 6.0 mmol/h) from the antacid table, which quickly increased the pH to neutral. FAM was immediately released from each unit after the dissolution of NaHCO_3_, and the *Q_FAM_* at 30 min was 89.7, 97.9, and 98.2%, respectively. The results show that the pH-responsive modular unit could deliver multiple drugs and could be assembled into a device for multiple self-regulation and combination therapies. The drug is released after acid neutralization, preventing the degradation of acid-unstable drugs, such as omeprazole and probiotics. 

As shown in Figure 8b, rapid acid secretion (labeled as a red arrow) at 0.5, 4.0, and 7.5 h triggered a rapid pH response in each unit (*T_rl_* = 7.0, 10.0, and 7.6 min). The pulsatile release of NaHCO_3_ (*V_m-NaHCO3_* = 7.5, 6.4, and 4.0 mmol/h) rapidly increased the pH to neutral values. FAM was sustained-released from each unit and completely released after 4 h (*Q_FAM_* = 94.6, 97.9, and 98.2%). During the three pH responses, FAM was released in a sustained manner for 12 h. The results in Figure 8a,b show that the pH-responsive modular unit loading immediate- or sustained-release preparation could be assembled into devices for multi-pulse or prolonged release. The combination of multiple drugs and multiple release behaviors facilitates the flexibility and personalization of treatment.

## 4. Conclusions

In this study, pH-responsive modular units were developed based on FDM-printed shells and assembled using soluble connectors and floating chambers. Each unit in the device could respond to excess acid secretion and release drugs independently. The drug release behavior of modular units can be predicted and customized on demand. Based on the assembly strategy, flexible and diverse pH-responsive DDSs can be developed for the personalized treatment of diseases with abnormal gastric acid secretion. General products for large numbers of patients can be printed in one step using a multi-nozzle 3D printer. 

For clinical safety and efficacy, systematic in vitro evaluation, such as content uniformity, hardness, floating duration, crystal form, and stability, should be further conducted. More bio-relevant medium containing buffers, penetrants, enzymes, and dietary ingredients [40] should also be used to investigate the actual performance (i.e., flotation performance, pH-responsiveness, and drug release behavior) of pH response devices in complex gastric environments. The application of floatation technologies for enhanced gastric retention is controversial, with in vivo studies not necessarily supporting their value [41]. However, continuing to explore this approach remains a valuable option for in vitro work as a preliminary to exploring if the approach in the current work might yet prove clinically useful. Furthermore, implementing this technology in clinic should help to overcome several barriers, for example, regulatory and safety issues, cost and scalability, and training and expertise [42].

## Data Availability

Data will be made available on request.

## References

[1] Di Mario F., Goni E. (2014). Gastric acid secretion: Changes during a century. Best Pract. Res. Clin. Gastroenterol..

[2] Lambert C.R., Varlotta D., Posey M., Heberlein J.L., Shirley J.M. (2015). Validation of the rightlevelph detector for monitoring. Am. J. Crit. Care.

[3] Fukuchi T., Ashida K., Yamashita H., Kiyota N., Tsukamoto R., Takahashi H., Ito D., Nagamatsu R. (2005). Influence of cure of Helicobacter pylori infection on gastric acidity and gastroesophageal reflux: Study by 24-h pH monitoring in patients with gastric or duodenal ulcer. J. Gastroenterol..

[4] Hwang K.M., Nguyen T.T., Seok S.H., Jo H.I., Cho C.H., Hwang K.M., Kim J.Y., Park C.W., Rhee Y.S., Park E.S. (2019). Swellable and porous bilayer tablet for gastroretentive drug delivery: Preparation and in vitro-in vivo evaluation. Int. J. Pharm..

[5] Cheng Z.J., Qing R., Hao S.L., Ding Y., Yin H.M., Zha G.D., Chen X.L., Ji J.O., Wang B.C. (2021). Fabrication of ulcer-adhesive oral keratin hydrogel for gastric ulcer healing in a rat. Regen. Biomater..

[6] Xu L.X., Bai E.H., Zhu Y.B., Qin J.Y., Du X., Huang H.Q. (2023). pH-responsive hydrogel as a potential oral delivery system of baicalin for prolonging gastroprotective activity. Pharmaceutics.

[7] Xu X., Liu H., Guo J.M., Huo Z.Y., Liu J., Wu Z.H., Qi X.L. (2020). Intragastric amorphous calcium carbonate consumption triggered generation of in situ hydrogel piece for sustained drug release. Int. J. Pharm..

[8] Jing H.J., Huang X., Du X.J., Mo L., Ma C.Y., Wang H.X. (2022). Facile synthesis of pH-responsive sodium alginate/carboxymethyl chitosan hydrogel beads promoted by hydrogen bond. Carbohydr. Polym..

[9] He J.H., Zhang Z.X., Yang Y.T., Ren F.G., Li J.P., Zhu S.J., Ma F., Wu R.Q., Lv Y., He G. (2021). Injectable self-healing adhesive pH-responsive hydrogels accelerate gastric hemostasis and wound healing. Nano-Micro Lett..

[10] Ding H.T., Tan P., Fu S.Q., Tian X.H., Zhang H., Ma X.L., Gu Z.W., Luo K. (2022). Preparation and application of pH-responsive drug delivery systems. J. Control. Release.

[11] Li X., Fu M., Wu J., Zhang C.Y., Deng X., Dhinakar A., Huang W.L., Qian H., Ge L. (2017). pH-sensitive peptide hydrogel for glucose-responsive insulin delivery. Acta Biomater..

[12] Tan P., Wu C.C., Tang Q., Wang T., Zhou C.L., Ding Y.K., Fu H.Y., Xu S.R., Feng Y.Q., Zhang Y.C. (2023). pH-triggered size-transformable and bioactivity-switchable self-assembling chimeric peptide nanoassemblies for combating drug-resistant bacteria and biofilms. Adv. Mater..

[13] Yoo O., Salman S., von Ungern-Sternberg B.S., Lim L.Y. (2023). Taste-masked flucloxacillin powder part 1: Optimisation of fabrication process using a mixture design approach. Pharmaceutics.

[14] Pradhan R., Kim S.Y., Yong C.S., Kim J.O. (2016). Preparation and characterization of spray-dried valsartan-loaded Eudragit^®^ EPO solid dispersion microparticles. Asian J. Pharm. Sci..

[15] Singh A.P., Siddiqui J., Diosady L.L. (2018). Characterizing the pH-dependent release kinetics of food-grade spray drying encapsulated iron microcapsules for food fortification. Food Bioprocess Technol..

[16] Goyanes A., Martinez P.R., Buanz A., Basit A.W., Gaisford S. (2015). Effect of geometry on drug release from 3D printed tablets. Int. J. Pharm..

[17] Chung S.Y., Zhang P.L., Repka M.A. (2023). Fabrication of timed-release indomethacin core-shell tablets for chronotherapeutic drug delivery using dual nozzle fused deposition modeling (FDM) 3D printing. Eur. J. Pharm. Biopharm..

[18] Tagami T., Nagata N., Hayashi N., Ogawa E., Fukushige K., Sakai N., Ozeki T. (2018). Defined drug release from 3D-printed composite tablets consisting of drug-loaded polyvinylalcohol and a water-soluble or water-insoluble polymer filler. Int. J. Pharm..

[19] Parulski C., Jennotte O., Lechanteur A., Evrard B. (2021). Challenges of fused deposition modeling 3D printing in pharmaceutical applications: Where are we now?. Adv. Drug Deliv. Rev..

[20] Okwuosa T.C., Soares C., Gollwitzer V., Habashy R., Timmins P., Alhnan M.A. (2018). On demand manufacturing of patient-specific liquid capsules via coordinated 3D printing and liquid dispensing. Eur. J. Pharm. Sci..

[21] Asadi M., Salehi Z., Akrami M., Hosseinpour M., Jockenhövel S., Ghazanfari S. (2023). 3D printed pH-responsive tablets containing N-acetylglucosamine-loaded methylcellulose hydrogel for colon drug delivery applications. Int. J. Pharm..

[22] Fu J.H., Yin H., Yu X., Xie C., Jiang H.L., Jin Y.G., Sheng F.G. (2018). Combination of 3D printing technologies and compressed tablets for preparation of riboflavin floating tablet-in-device (TiD) systems. Int. J. Pharm..

[23] Charoenying T., Patrojanasophon P., Ngawhirunpat T., Rojanarata T., Akkaramongkolporn P., Opanasopit P. (2020). Three-dimensional (3D)-printed devices composed of hydrophilic cap and hydrophobic body for improving buoyancy and gastric retention of domperidone tablets. Eur. J. Pharm. Sci..

[24] Tagami T., Hayashi N., Sakai N., Ozeki T. (2019). 3D printing of unique water-soluble polymer-based suppository shell for controlled drug release. Int. J. Pharm..

[25] Khaled S.A., Burley J.C., Alexander M.R., Yang J., Roberts C.J. (2015). 3D printing of five-in-one dose combination polypill with defined immediate and sustained release profiles. J. Control. Release.

[26] Genina N., Boetker J.P., Colombo S., Harmankaya N., Rantanen J., Bohr A. (2017). Anti-tuberculosis drug combination for controlled oral delivery using 3D printed compartmental dosage forms: From drug product design to in vivo testing. J. Control. Release.

[27] Tan Y.J.N., Yong W.P., Kochhar J.S., Khanolkar J., Yao X.K., Sun Y.J., Ao C.K., Soh S. (2020). On-demand fully customizable drug tablets via 3D printing technology for personalized medicine. J. Control. Release.

[28] Matijasic G., Gretic M., Vincic J., Poropat A., Cuculic L., Rahelic T. (2019). Design and 3D printing of multi-compartmental PVA capsules for drug delivery. J. Drug Deliv. Sci. Technol..

[29] Maroni A., Melocchi A., Parietti F., Foppoli A., Zema L., Gazzaniga A. (2017). 3D printed multi-compartment capsular devices for two-pulse oral drug delivery. J. Control. Release.

[30] Melocchi A., Uboldi M., Parietti F., Cerea M., Foppoli A., Palugan L., Gazzaniga A., Maroni A., Zema L. (2020). Lego-inspired capsular devices for the development of personalized dietary supplements: Proof of concept with multimodal release of caffeine. J. Pharm. Sci..

[31] Kirtane A.R., Abouzid O., Minahan D., Bensel T., Hill A.L., Selinger C., Bershteyn A., Craig M., Mo S.S., Mazdiyasni H. (2018). Development of an oral once-weekly drug delivery system for HIV antiretroviral therapy. Nat. Commun..

[32] Bellinger A.M., Jafari M., Grant T.M., Zhang S.Y., Slater H.C., Wenger E.A., Mo S., Lee Y.A.L., Mazdiyasni H., Kogan L. (2016). Oral, ultra-long-lasting drug delivery: Application toward malaria elimination goals. Sci. Transel. Med..

[33] Myung N., Jin S., Cho H.J., Kang H.W. (2022). User-designed device with programmable release profile for localized treatment. J. Control. Release.

[34] Alqahtani A.A., Mohammed A.A., Fatima F., Ahmed M.M. (2023). Fused deposition modelling 3D-printed gastro-retentive floating device for propranolol hcl tablets. Polymers.

[35] Garbacz G., Kolodziej B., Koziolek M., Weitschies W., Klein S. (2014). A dynamic system for the simulation of fasting luminal pH-gradients using hydrogen carbonate buffers for dissolution testing of ionisable compounds. Eur. J. Pharm. Sci..

[36] Scott N., Patel K., Sithole T., Xenofontos K., Mohylyuk V., Liu F. (2020). Regulating the pH of bicarbonate solutions without purging gases: Application to dissolution testing of enteric coated tablets, pellets and microparticles. Int. J. Pharm..

[37] Yang Y., Wang Y.M., Li J., Pan W.S. (2014). Manufacture and characteristics of asymmetric membrane capsule shells with a novel wet phase inversion method. Drug Dev. Ind. Pharm..

[38] Zhao X., Wei W., Niu R., Li Q., Hu C., Jiang S. (2022). 3D printed intragastric floating and sustained-release tablets with air chambers. J. Pharm. Sci..

[39] Charoenying T., Opanasopit P., Ngawhirunpat T., Rojanarata T., Akkaramongkolporn P., Patrojanasophon P. (2023). Development of a novel tablet-shaped floating 3D-printed device with adjustable floating time as floating drug delivery systems provided zero-order release kinetics. J. Drug Deliv. Sci. Technol..

[40] Litou C., Vertzoni M., Xu W., Kesisoglou F., Reppas C. (2017). The impact of reduced gastric acid secretion on dissolution of salts of weak bases in the fasted upper gastrointestinal lumen: Data in biorelevant media and in human aspirates. Eur. J. Pharm. Biopharm..

[41] Grimm M., Ball K., Scholz E., Schneider F., Sivert A., Benameur H., Kromrey M.L., Kühn J.P., Weitschies W. (2019). Characterization of the gastrointestinal transit and disintegration behavior of floating and sinking acid-resistant capsules using a novel MRI labeling technique. Eur. J. Pharm. Sci..

[42] Huanbutta K., Burapapadh K., Sriamornsak P., Sangnim T. (2023). Practical application of 3D printing for pharmaceuticals in hospitals and pharmacies. Pharmaceutics.

